# Evaluating objective nutritional biomarkers for the prediction of sarcopenia in Crohn’s disease: development and internal validation of a clinical nomogram

**DOI:** 10.3389/fnut.2026.1838106

**Published:** 2026-07-09

**Authors:** Hai-yuan Zhong, Wen-tao Deng, Jing-shuai Huang, Jin-cheng Li, Xiao-ling Luo, Chun-mei Liang, Guang Xiong, Ming-yu Lai

**Affiliations:** 1 Department of Gastroenterology, The First Affiliated Hospital of Guangxi Medical University, Nanning, China; 2 Department of Geriatric Endocrinology and Metabolism, The First Affiliated Hospital of Guangxi Medical University, Nanning, China

**Keywords:** advanced lung cancer inflammation index, Crohn’s disease, geriatric nutritional risk index, nomogram, predictive model, prognostic nutritional index, sarcopenia

## Abstract

**Background:**

Sarcopenia is prevalent in Crohn’s disease (CD) and correlates with adverse clinical outcomes; however, routine computed tomography (CT) screening is limited in daily practice. This study aimed to comparatively analyze three objective nutritional indices—the Geriatric Nutritional Risk Index (GNRI), Advanced Lung Cancer Inflammation Index (ALI), and Prognostic Nutritional Index (PNI)—and to develop a predictive nomogram for sarcopenia in CD.

**Methods:**

A retrospective cohort of 333 patients with CD was randomly allocated into a training (*n* = 234) and an internal validation (*n* = 99) cohort. Sarcopenia was defined using gender-specific cut-offs of the CT-derived skeletal muscle index (SMI) at the third lumbar vertebra (L3) level. Unadjusted restricted cubic splines (RCS) evaluated continuous associations. Multivariable logistic regression was utilized to identify associated risk factors and construct the nomogram. Model performance was assessed via the area under the receiver operating characteristic curve (AUC), calibration plots, and decision curve analysis (DCA). Interaction tests evaluated prognostic stability.

**Results:**

The overall prevalence of sarcopenia was 48.3%. All three indices demonstrated linear inverse continuous associations with sarcopenia risk and maintained robust statistical associations after multivariable adjustment. The GNRI demonstrated comparable discriminative accuracy to the ALI, significantly outperformed the PNI, and remained highly stable across all clinical subgroups (all P for interaction > 0.05). Conversely, the predictive capacities of the ALI and PNI were significantly confounded by disease activity. The formulated nomogram, integrating the GNRI with sex, Montreal location, Montreal behavior, and disease activity, exhibited excellent discrimination (training AUC = 0.847; validation AUC = 0.750). Calibration plots indicated optimal agreement, and DCA confirmed maximum net clinical benefit for the GNRI-based model.

**Conclusion:**

The GNRI is a robust surrogate biomarker for identifying sarcopenia in CD, demonstrating excellent predictive performance comparable to the ALI, alongside high clinical stability. The developed GNRI-based nomogram provides an accurate, non-invasive screening tool to facilitate early identification and guide targeted nutritional interventions.

## 1 Introduction

Crohn’s disease (CD) is a chronic inflammatory bowel disease characterized by transmural gastrointestinal inflammation. Driven by systemic inflammation, impaired nutrient absorption, and increased metabolic demands, patients with CD frequently experience severe malnutrition (1–5). This nutritional decline often manifests as sarcopenia, defined by the progressive loss of skeletal muscle mass. Recent evidence indicates that sarcopenia is not merely a consequence of active inflammation but an independent prognostic factor for adverse outcomes in CD, including higher surgical rates, postoperative complications, and prolonged hospital stays (6–10). Consequently, early identification of sarcopenia is critical for the comprehensive management of these patients.

Currently, cross-sectional imaging, particularly computed tomography (CT) at the third lumbar vertebra (L3), serves as the reference standard for quantifying skeletal muscle mass (11–13). Additionally, dual-energy X-ray absorptiometry (DEXA) is widely recognized as a useful and accessible tool for evaluating body composition in general clinical practice. However, in the setting of hospitalized patients with CD, abdominal CT scans are routinely performed for diagnostic purposes to evaluate disease extent and rule out penetrating complications, such as abscesses or fistulas. Utilizing these pre-existing CT images for opportunistic muscle mass assessment provides highly accurate anatomical resolution while avoiding the need for additional DEXA scans, thereby sparing patients from extra healthcare costs and logistical burdens. Nevertheless, the routine application of CT strictly for continuous nutritional screening in outpatient settings is limited by cumulative radiation exposure, substantial healthcare costs, and the necessity for specialized analytical software (14). These operational constraints highlight an unmet clinical need for simple, non-invasive, and cost-effective surrogate biomarkers applicable in daily outpatient or ward settings.

Several objective, blood-based nutritional and inflammatory indices, such as the Geriatric Nutritional Risk Index (GNRI), Advanced Lung Cancer Inflammation Index (ALI), and Prognostic Nutritional Index (PNI), have been proposed as practical alternatives. These indices are derived from readily available anthropometric and laboratory parameters, including body mass index (BMI), serum albumin, and lymphocyte counts, and are used to reflect a patient’s immunonutritional status. Each index has demonstrated strong prognostic value in different clinical settings. The GNRI has been extensively validated as a reliable predictor of morbidity and mortality in geriatric populations, as well as in patients with chronic heart failure and end-stage renal disease (15–18). The ALI, originally developed in the field of oncology, shows high accuracy in assessing systemic inflammation and cachexia in patients with advanced malignancies (19–21). In addition, the PNI is widely used for surgical risk stratification and for predicting postoperative complications, particularly in gastrointestinal cancers and cardiovascular surgery (22, 23). Although these indices have been successfully applied in their respective fields, their ability to specifically reflect skeletal muscle depletion in patients with Crohn’s disease remains insufficiently investigated.

Despite the overlapping pathophysiology of systemic malnutrition and muscle wasting, no head-to-head comparative analysis has evaluated the optimal predictive index for sarcopenia in patients with CD. Furthermore, a practical tool integrating such an index with routine clinical parameters is lacking. Therefore, this study aimed to: (1) comprehensively compare the predictive performance of GNRI, ALI, and PNI for CT-defined sarcopenia in a well-defined CD cohort; (2) characterize the continuous associations between varying levels of these indices and the risk of sarcopenia using restricted cubic spline analysis, as well as evaluate their prognostic stability across different clinical phenotypes; and (3) develop and internally validate an index-based clinical nomogram. We hypothesized that identifying the most robust nutritional index could yield a reliable screening tool to facilitate early, targeted interventions.

## 2 Materials and methods

### 2.1 Study design and participants

**Figure 1:**
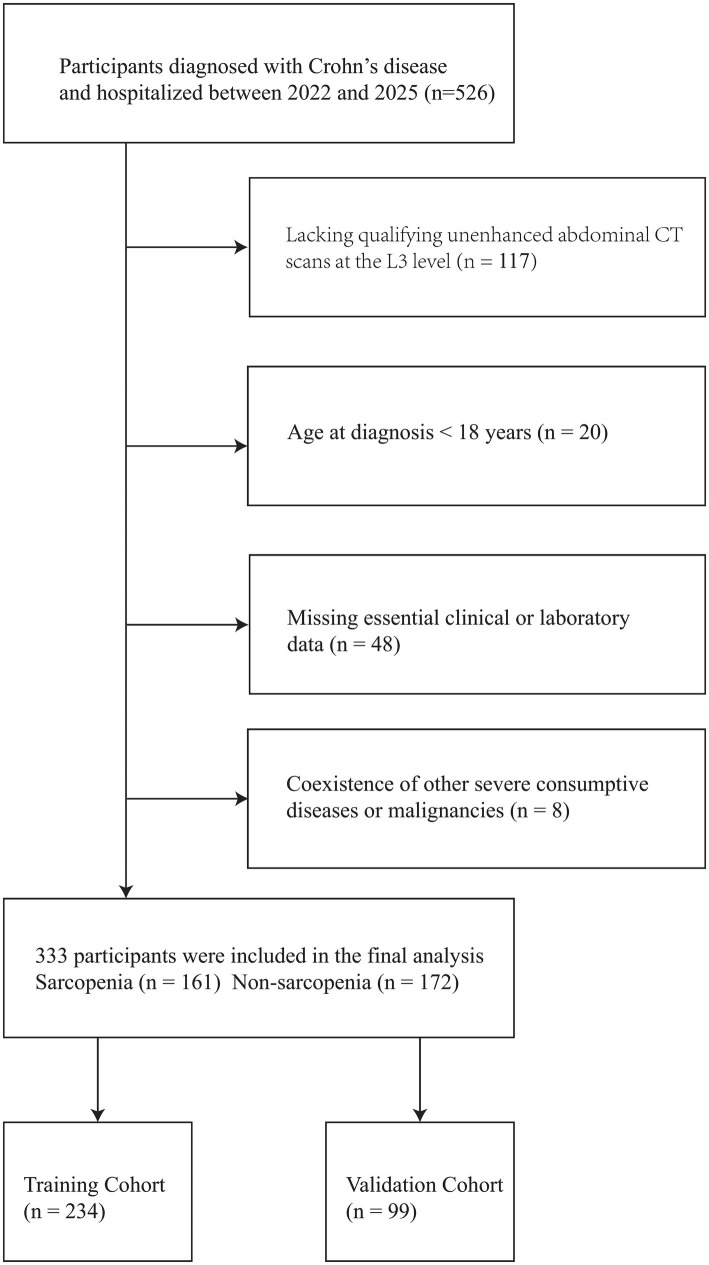
Flowchart of patient selection and study design.

This retrospective cohort study was conducted at the First Affiliated Hospital of Guangxi Medical University. We consecutively screened patients diagnosed with Crohn’s disease (CD) who were hospitalized between 2022 and 2025 (Figure 1). The study protocol was approved by the Institutional Review Board (IRB) of the First Affiliated Hospital of Guangxi Medical University. Given the retrospective nature of the study and the use of anonymized clinical data, the requirement for written informed consent was waived.

To ensure cohort homogeneity and the accuracy of body composition analysis, patients were strictly evaluated against the following inclusion criteria: (1) definitive diagnosis of CD based on standard clinical, endoscopic, radiological, and histological consensus criteria; (2) age ≥ 18 years, ensuring complete skeletal muscle development; (3) availability of high-quality unenhanced abdominal computed tomography (CT) scans encompassing the entire third lumbar vertebra (L3) level during hospitalization; and (4) completeness of essential baseline clinical and laboratory data.

To rigorously eliminate confounding factors affecting skeletal muscle mass, the exclusion criteria were predefined as follows: (1) coexistence of other severe consumptive diseases contributing to non-CD-related muscle loss, including malignancies, Child-Pugh C hepatic insufficiency, an estimated glomerular filtration rate below 30 mL/min, hyperthyroidism, or active tuberculosis; (2) neuromuscular disorders inducing neurogenic muscle atrophy, such as myasthenia gravis or multiple sclerosis; (3) a history of prolonged bed rest or limb immobilization exceeding 2 weeks prior to admission, leading to disuse atrophy; (4) pregnancy or lactation; (5) severe CT image artifacts caused by metallic implants or patient motion, or incomplete L3 anatomical structures precluding the accurate delineation of muscle boundaries; (6) a time interval exceeding 7 days between the abdominal CT acquisition and laboratory blood tests, to prevent temporal confounding; and (7) recent major abdominal surgery within 4 weeks prior to the baseline assessment, to avoid the acute confounding effects of surgical stress on systemic inflammatory markers, nutritional parameters, and rapid muscle catabolism. Additionally, patients with a history of prior CD-related intestinal surgery (performed > 4 weeks prior) were included, and this variable was adjusted for in the subsequent baseline and regression analyses.

### 2.2 Clinical and laboratory data collection

Demographic and clinical characteristics, including sex, age, body mass index (BMI), smoking and drinking history, history of intestinal surgery, and perianal complications, were extracted from electronic medical records. The disease phenotype was classified according to the Montreal classification (age at diagnosis, disease location, and disease behavior). Disease activity was evaluated using the Harvey-Bradshaw Index (HBI) and categorized as mild (≤ 6), moderate (7–15), or severe (≥ 16) (24).

Laboratory parameters, including white blood cell (WBC) count, hemoglobin, platelets, neutrophil count, lymphocyte count, total bilirubin, and serum albumin, were collected within 7 days of the CT scan. The three nutritional indices were calculated using the following formulas.

First, the ideal body weight (IBW) was calculated using the Lorentz equation:


$$\mathrm{IB}W_{\text{male}}=\text{Height}-100-\frac{\text{Height}-150}{4}$$

$$\mathrm{IB}W_{\text{female}}=\text{Height}-100-\frac{\text{Height}-150}{2.5}$$

Subsequently, the indices were defined as:


$$\text{GNRI}=1.489\times \text{Albumin}\;(g/L)+41.7\times \frac{\text{Weight}}{\mathrm{IBW}}$$

$$\mathrm{ALI}=\frac{\mathrm{BMI}\times \text{Albumin}(g/\mathrm{dL})}{\mathrm{NLR}}$$

PNI=10×Albumin(g/dL)+0.005×Total Lymphocyte Count(/μL)

(Note: NLR represents the neutrophil-to-lymphocyte ratio; albumin is measured in g/L for GNRI and g/dL for ALI/PNI; lymphocytes are measured per mm^3^).

To facilitate clinical interpretation and statistical modeling, several baseline characteristics were transformed into categorical variables. According to the Montreal classification, disease location was dichotomized into the L3 (ileocolonic) versus non-L3 (ileal or colonic) phenotype. Disease behavior was classified as B1 (non-stricturing, non-penetrating) versus non-B1 (stricturing [B2] or penetrating [B3]). Smoking history, drinking history, previous intestinal surgery, and the presence of perianal complications were recorded as binary variables (Yes/No). Furthermore, to evaluate potential non-linear continuous associations and ceiling effects, the continuous values of the GNRI, ALI, and PNI were converted into categorical variables based on their respective tertiles (T1, lowest; T2, middle; T3, highest). The lowest tertile (T1) served as the reference group in subsequent regression models.

### 2.3 Image analysis and definition of sarcopenia

**Figure 2:**
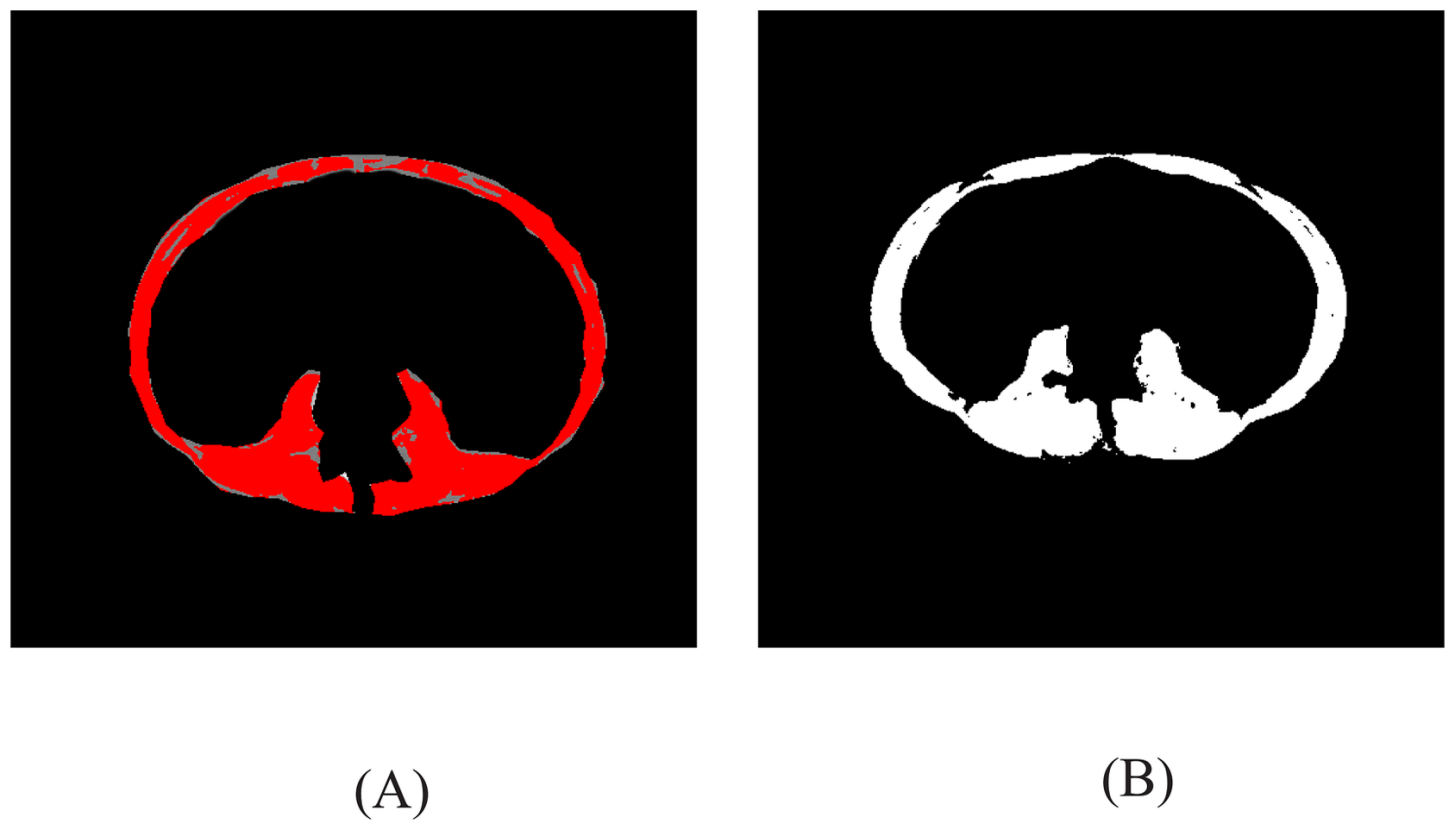
Semi-automatic segmentation of skeletal muscle area at the third lumbar vertebra (L3) level. **(A)** CT image of a patient with sarcopenia, showing reduced muscle mass (red area). **(B)** CT image of a patient without sarcopenia, showing preserved muscle mass (white area).

Skeletal muscle mass was evaluated using cross-sectional unenhanced abdominal CT images at the level of the third lumbar vertebra (L3). The skeletal muscle area (SMA, cm^2^), encompassing the psoas, paraspinal, and abdominal wall muscles, was semi-automatically quantified using ImageJ software based on standard Hounsfield unit (HU) thresholds (−29 to +150 HU; Figure 2). To minimize measurement bias, radiological evaluations were independently performed by two experienced gastroenterologists blinded to the patients’ clinical outcomes. The skeletal muscle index (SMI, cm^2^/m^2^) was calculated by normalizing the SMA by the square of the patient’s height.

According to specific diagnostic criteria derived from large-scale Chinese cohorts, sarcopenia was defined as an SMI < 44.77 cm^2^/m^2^ for males and < 32.5 cm^2^/m^2^ for females (25).

### 2.4 Statistical analysis

All statistical analyses were performed using R software (version 4.4.3). Continuous variables were presented as mean ± standard deviation (SD) and compared using the Student’s t-test or Mann–Whitney U test, as appropriate. Categorical variables were expressed as frequencies (percentages) and analyzed using the Chi-square test or Fisher’s exact test.

To visualize the functional forms of these relationships, unadjusted restricted cubic splines (RCS) with three knots were employed to assess the raw continuous associations between the nutritional indices and sarcopenia risk. Additionally, multivariable linear regression was performed to assess the association between the indices and actual continuous SMI values.

In the training cohort, univariable and multivariable logistic regression analyses were performed to identify associated risk factors for sarcopenia. The variable selection for the final multivariable model and the subsequent nomogram followed a predefined three-step approach to ensure reproducibility and clinical applicability. First, variables demonstrating statistical significance (*p* < 0.05) in the univariable analysis were identified as primary candidates. Second, to minimize severe multicollinearity, variables that are intrinsic components of the evaluated nutritional indices, including body mass index, serum albumin, neutrophil count, and lymphocyte count, were strictly excluded. Finally, from the remaining candidates, variables with well-established clinical relevance to Crohn’s disease progression and nutritional deterioration, specifically sex, Montreal location, Montreal behavior, and disease activity, were incorporated into the final multivariable model to develop the predictive nomogram. Model performance was assessed using the area under the receiver operating characteristic curve (AUC), and DeLong’s test was used for pairwise comparisons of ROC curves. Calibration of the nomogram was evaluated using calibration plots. In addition, decision curve analysis (DCA) was conducted to assess the clinical utility and net benefit of the models. Subgroup analyses and interaction tests were further performed to examine the robustness of the indices across different clinical strata. A two-sided *p* value < 0.05 was considered statistically significant.

## 3 Result

### 3.1 Baseline characteristics of the study population

A flowchart detailing the patient screening and inclusion process is presented in Figure 1. The final analysis included 333 eligible patients with Crohn’s disease (CD), among whom 161 (48.3%) were diagnosed with sarcopenia. The cohort was randomly allocated into a training cohort (*n* = 234) and an internal validation cohort (*n* = 99). Baseline demographic, clinical, and laboratory features were well-balanced between the two cohorts (all *p* > 0.05, Supplementary Table 1).

Detailed baseline characteristics stratified by the presence of sarcopenia are summarized in Table 1. Demographically, patients with sarcopenia were younger (32.73 ± 11.63 vs. 36.86 ± 11.38 years, *p* = 0.001), had a significantly lower body mass index (BMI; 16.99 ± 2.32 vs. 20.96 ± 3.24 kg/m^2^, *p* < 0.001), and were predominantly male (78.9% vs. 59.3%, *p* < 0.001). Regarding disease phenotypes based on the Montreal classification, the sarcopenia group presented a higher prevalence of the A2 phenotype (17–40 years at diagnosis; 74.5% vs. 59.9%, *p* = 0.006) and non-B1 behavior (structuring or penetrating disease; 50.9% vs. 38.4%, *p* = 0.028). Additionally, sarcopenic patients exhibited more severe disease activity (*p* = 0.002) and a higher incidence of perianal complications (31.1% vs. 19.8%, *p* = 0.025). However, no significant differences were observed regarding Montreal location, smoking history, drinking history, or previous intestinal surgery (all *p* > 0.05).

**Table 1:** Baseline characteristics of patients with Crohn’s disease stratified by sarcopenia.

Characteristics	Total (*N* = 333)	Non-sarcopenia (*n* = 172)	Sarcopenia (*n* = 161)	*p*-value
Sex, n (%)				<0.001
Male	229 (68.8)	102 (59.3)	127 (78.9)	
Female	104 (31.2)	70 (40.7)	34 (21.1)	
Age (years)	34.86 ± 11.66	36.86 ± 11.38	32.73 ± 11.63	0.001
BMI (kg/m^2^)	19.04 ± 3.45	20.96 ± 3.24	16.99 ± 2.32	<0.001
Montreal age				0.006
A2	223 (67.0)	103 (59.9)	120 (74.5)	
A3	110 (33.0)	69 (40.1)	41 (25.5)	
Montreal location, n (%)				0.086
L3	213 (64.0)	102 (59.3)	111 (68.9)	
non-L3	120 (36.0)	70 (40.7)	50 (31.1)	
Montreal behavior, n (%)				0.028
B1	185 (55.6)	106 (61.6)	79 (49.1)	
non-B1	148 (44.4)	66 (38.4)	82 (50.9)	
Disease activity, n (%)				0.002
Mild	28 (8.4)	21 (12.2)	7 (4.3)	
Moderate	284 (85.3)	146 (84.9)	138 (85.7)	
Severe	21 (6.3)	5 (2.9)	16 (9.9)	
Smoking history, n (%)				1
No	248 (74.5)	128 (74.4)	120 (74.5)	
Yes	85 (25.5)	44 (25.6)	41 (25.5)	
Drinking history, n (%)				0.332
No	256 (76.9)	128 (74.4)	128 (79.5)	
Yes	77 (23.1)	44 (25.6)	33 (20.5)	
Surgery history, n (%)				0.438
No	292 (87.7)	148 (86.0)	144 (89.4)	
Yes	41 (12.3)	24 (14.0)	17 (10.6)	
Perianal complication, n (%)				0.025
No	249 (74.8)	138 (80.2)	111 (68.9)	
Yes	84 (25.2)	34 (19.8)	50 (31.1)	
WBC (×10^9^/L)	8.05 ± 3.78	7.96 ± 3.94	8.14 ± 3.60	0.662
Hemoglobin (g/L)	113.28 ± 24.40	118.38 ± 22.14	107.82 ± 25.55	<0.001
Platelets (×10^9^/L)	374.79 ± 137.84	344.39 ± 111.35	407.26 ± 155.31	<0.001
Neutrophils (×10^9^/L)	5.77 ± 3.59	5.42 ± 3.81	6.13 ± 3.31	0.07
Lymphocytes (×10^9^/L)	1.38 ± 0.60	1.56 ± 0.63	1.20 ± 0.51	<0.001
Total bilirubin (umol/L)	8.54 ± 10.66	9.39 ± 14.24	7.64 ± 4.17	0.134
Albumin (g/L)	37.31 ± 6.60	39.22 ± 5.85	35.28 ± 6.76	<0.001
SMI (cm^2^/m^2^)	41.16 ± 8.64	45.91 ± 7.84	36.09 ± 6.26	<0.001
GNRI	90.38 ± 12.49	96.20 ± 10.38	84.16 ± 11.54	<0.001
GNRI_T, n (%)				<0.001
T1	111 (33.3)	24 (14.0)	87 (54.0)	
T2	111 (33.3)	60 (34.9)	51 (31.7)	
T3	111 (33.3)	88 (51.2)	23 (14.3)	
ALI	24.70 ± 27.92	33.62 ± 35.05	15.16 ± 11.30	<0.001
ALI_T, n (%)				<0.001
T1	111 (33.3)	30 (17.4)	81 (50.3)	
T2	111 (33.3)	54 (31.4)	57 (35.4)	
T3	111 (33.3)	88 (51.2)	23 (14.3)	
PNI	44.23 ± 7.93	47.00 ± 6.92	41.26 ± 7.87	<0.001
PNI_T, n (%)				<0.001
T1	111 (33.3)	30 (17.4)	81 (50.3)	
T2	111 (33.3)	66 (38.4)	45 (28.0)	
T3	111 (33.3)	76 (44.2)	35 (21.7)	

Continuous variables are presented as mean ± standard deviation (SD), and categorical variables are presented as frequencies and percentages (%). *p* values were calculated using the Student’s t-test for continuous variables and the Chi-square test for categorical variables. BMI, Body Mass Index; WBC, White Blood Cell; SMI, Skeletal Muscle Index; GNRI, Geriatric Nutritional Risk Index; ALI, Advanced Lung Cancer Inflammation Index; PNI, Prognostic Nutritional Index.

Laboratory assessments revealed that the sarcopenia group had significantly lower levels of hemoglobin, lymphocytes, and serum albumin, alongside elevated platelet counts (all *p* < 0.001). Conversely, white blood cell (WBC) counts, neutrophils, and total bilirubin levels were comparable between the two groups (all *p* > 0.05). The actual skeletal muscle index (SMI) was substantially diminished in the sarcopenia group (36.09 ± 6.26 vs. 45.91 ± 7.84 cm^2^/m^2^, *p* < 0.001). Consistently, the continuous values and categorical tertile distributions of all three nutritional indices (GNRI, ALI, and PNI) were significantly lower in patients with sarcopenia (all *p* < 0.001).

**Figure 3:**
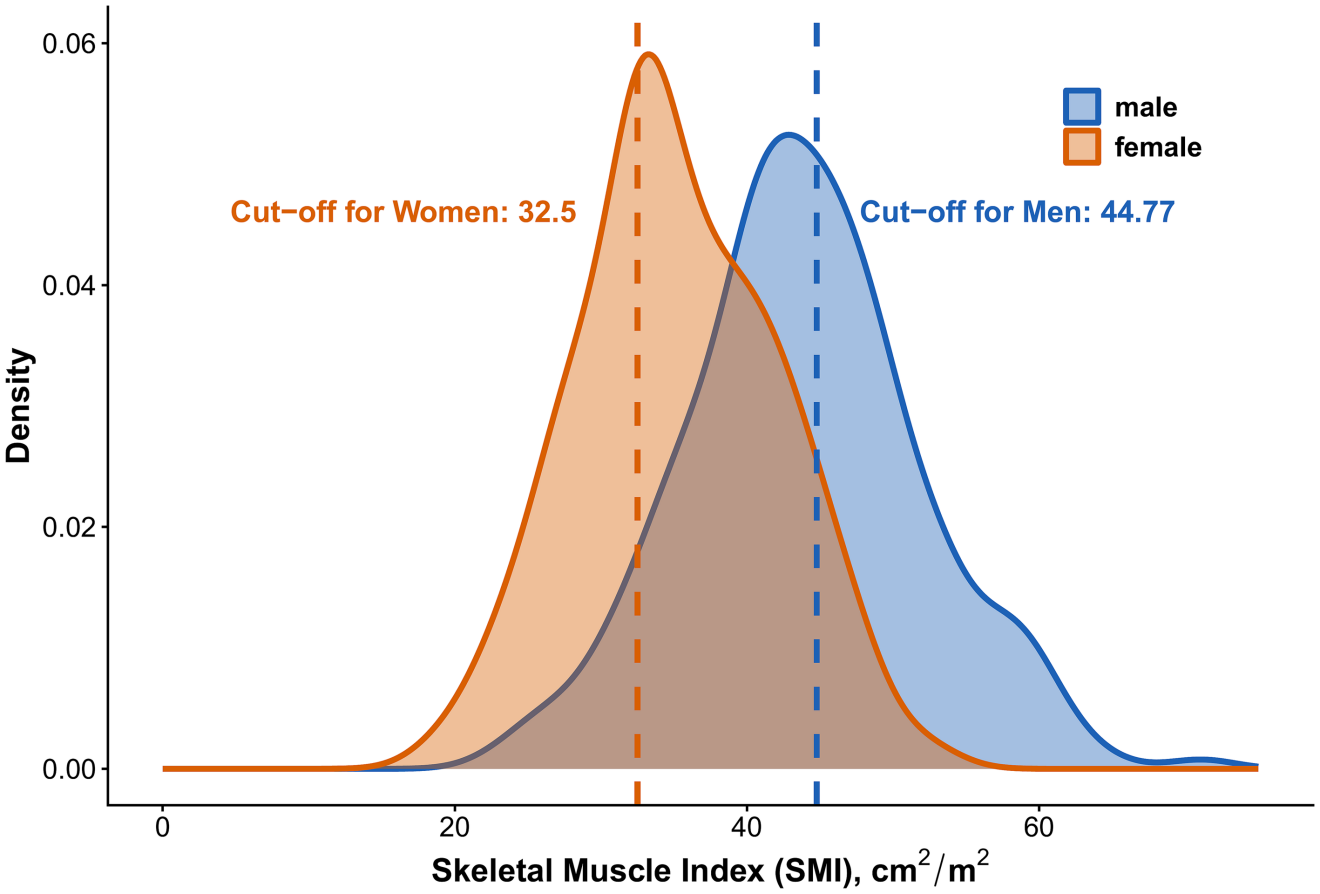
Density distribution of skeletal muscle index (SMI) stratified by sex. The vertical dashed lines represent the gender-specific diagnostic cut-off values for sarcopenia (44.77 cm^2^/m^2^ for males, blue, 32.5 cm^2^/m^2^ for females, orange).

The density distribution of SMI across the overall cohort is visually depicted in Figure 3. By applying gender-specific cut-offs (44.77 cm^2^/m^2^ for males, 32.5 cm^2^/m^2^ for females), a marked proportion of patients (falling to the left of the diagnostic threshold lines) were identified with muscle depletion, illustrating the substantial burden of sarcopenia in this CD population.

### 3.2 Continuous associations and functional forms

**Figure 4:**
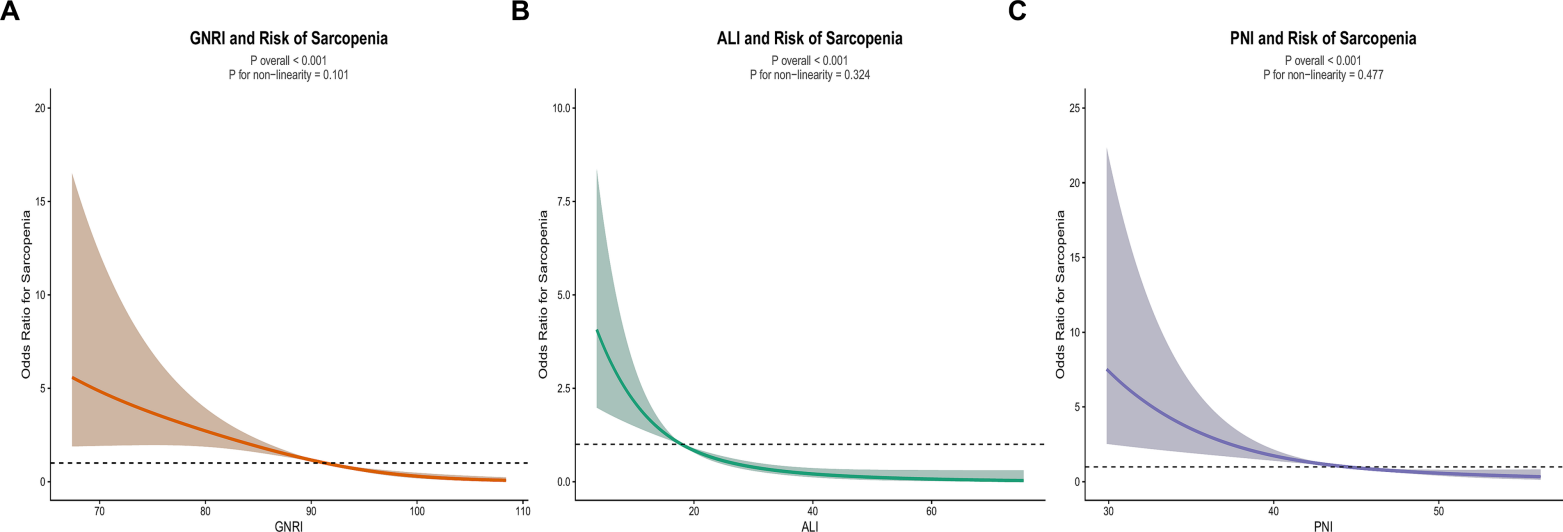
Restricted cubic spline (RCS) curves illustrating the continuous associations between nutritional indices and the risk of sarcopenia. **(A)** GNRI; **(B)** ALI; **(C)** PNI. These spline models are unadjusted to illustrate the raw functional forms.

To evaluate the functional forms of the associations between the nutritional indices and sarcopenia risk, unadjusted restricted cubic spline (RCS) analyses were conducted (Figure 4). The models confirmed a highly significant overall association for all three indices (all P overall < 0.001). Statistical tests for non-linearity indicated that these relationships were essentially linear (GNRI: P for non-linearity = 0.101; ALI: P for non-linearity = 0.324; PNI: P for non-linearity = 0.477). The plotted curves display a continuous, monotonic protective pattern, where higher values of GNRI, ALI, and PNI correspond to progressively decreased odds ratios (ORs) for sarcopenia, thereby justifying their inclusion as continuous or ordinal variables in subsequent models.

### 3.3 Multivariable analysis of associated risk factors

Univariate logistic regression analysis in the training cohort (Table 2) identified female sex, non-L3 location, non-B1 behavior, moderate-to-severe disease activity, lower lymphocyte counts, lower total bilirubin, and lower albumin levels as significant risk factors for sarcopenia. In unadjusted models, higher GNRI, ALI, and PNI scores were strongly protective against sarcopenia (all *p* < 0.001).

**Table 2:** Univariate analysis of factors associated with sarcopenia in patients with Crohn’s disease.

Variables	Total (n)	Event (n)	OR (95% CI)	*p*-value
Sex				
Male	159	88	-	-
Female	75	22	0.335 (0.183–0.596)	**<0.001**
Montreal age				
A2 (17–40 years)	155	76	-	-
A3 (>40 years)	79	34	0.785 (0.453–1.353)	0.385
Montreal location				
L3	150	79	-	-
non-L3	84	31	0.526 (0.302–0.904)	**0.021**
Montreal behavior				
B1	128	51	-	-
non-B1	106	59	1.895 (1.128–3.206)	**0.016**
Disease activity				
Mild	21	4	-	-
Moderate	196	92	3.760 (1.335–13.424)	**0.021**
Severe	17	14	19.833 (4.280–124.458)	**<0.001**
Smoking history				
No	175	80	-	-
Yes	59	30	1.228 (0.680–2.224)	0.495
Drinking history				
No	177	83	-	-
Yes	57	27	1.019 (0.559–1.854)	0.95
Intestinal surgery history				
No	202	97	-	-
Yes	32	13	0.741 (0.340–1.568)	0.437
Perianal complication				
No	171	76	-	-
Yes	63	34	1.466 (0.821–2.629)	0.197
White blood cell	234	110	1.007 (0.942–1.078)	0.834
Hemoglobin	234	110	0.980 (0.969–0.990)	**<0.001**
Platelet	234	110	1.004 (1.002–1.007)	**<0.001**
Neutrophil	234	110	1.059 (0.987–1.147)	0.13
Lymphocyte	234	110	0.228 (0.126–0.391)	**<0.001**
Total bilirubin	234	110	0.933 (0.876–0.987)	**0.025**
Albumin	234	110	0.896 (0.854–0.937)	**<0.001**
GNRI	234	110	0.901 (0.873–0.927)	**<0.001**
ALI	234	110	0.925 (0.900–0.948)	**<0.001**
PNI	234	110	0.889 (0.852–0.925)	**<0.001**

OR, Odds Ratio; CI, Confidence Interval; GNRI, Geriatric Nutritional Risk Index; ALI, Advanced Lung Cancer Inflammation Index; PNI, Prognostic Nutritional Index. The bold values indicate statistical significance (*p* < 0.05).

To evaluate the adjusted predictive capacity of these indices, multivariable logistic regression models were developed (Table 3). After full adjustment for demographic, clinical, and laboratory confounders (Model 3), GNRI, ALI, and PNI maintained robust statistically significant inverse associations with sarcopenia when analyzed as continuous variables (all *p* < 0.001). However, considering that these indices are composite measures incorporating fundamental nutritional and inflammatory parameters, these statistical associations should be interpreted as an integrated reflection of the patients’ overarching nutritional-inflammatory status, rather than absolute conceptual independence from the muscle depletion process. When evaluated as tertiles, a dose-dependent protective trend was confirmed. Notably, while the highest tertiles (T3) of GNRI and ALI showed substantial risk reductions compared to T2, the protective effect of PNI plateaued, with the OR in T3 (0.178) showing limited incremental improvement over T2 (0.218).

**Table 3:** Association between nutritional indices and the risk of sarcopenia in patients with Crohn’s disease.

Variables		Model 1		Model 2		Model 3	
		OR (95% CI)	*p-*value	OR (95% CI)	*p-*value	OR (95% CI)	*p-*value
GNRI	Continuous (per 1 unit)	0.901 (0.873, 0.927)	<0.001	0.895 (0.864, 0.924)	<0.001	0.889 (0.849, 0.927)	<0.001
	T1(ref)	-	-	-	-	-	-
	T2	0.157 (0.074, 0.317)	<0.001	0.138 (0.058, 0.308)	<0.001	0.148 (0.057, 0.363)	<0.001
	T3	0.061 (0.027, 0.131)	<0.001	0.059 (0.023, 0.138)	<0.001	0.073 (0.023, 0.219)	<0.001
	*P* for trend		<0.001		<0.001		<0.001
ALI	Continuous (per 1 unit)	0.925 (0.900, 0.948)	<0.001	0.924 (0.895, 0.949)	<0.001	0.915 (0.879, 0.947)	<0.001
	T1(ref)	-	-	-	-	-	-
	T2	0.408 (0.207, 0.791)	0.009	0.380 (0.179, 0.789)	0.01	0.294 (0.119, 0.689)	0.006
	T3	0.067 (0.029, 0.144)	<0.001	0.066 (0.026, 0.154)	<0.001	0.062 (0.020, 0.174)	<0.001
	*P* for trend		<0.001		<0.001		<0.001
PNI	Continuous (per 1 unit)	0.889 (0.852, 0.925)	<0.001	0.885 (0.844, 0.925)	<0.001	0.895 (0.842, 0.949)	<0.001
	T1(ref)	-	-	-	-	-	-
	T2	0.216 (0.107, 0.421)	<0.001	0.182 (0.080, 0.395)	<0.001	0.218 (0.087, 0.516)	<0.001
	T3	0.135 (0.065, 0.270)	<0.001	0.120 (0.052, 0.264)	<0.001	0.178 (0.061, 0.490)	0.001
	*P* for trend		<0.001		<0.001		0.001

Model 1: Crude model, unadjusted. Model 2: Adjusted for sex, Montreal age at diagnosis, Montreal location, Montreal behavior, disease activity, smoking history, drinking history, history of intestinal surgery, and perianal complications. Model 3: Adjusted for sex, Montreal age at diagnosis, Montreal location, Montreal behavior, disease activity, smoking history, drinking history, history of intestinal surgery, perianal complications, hemoglobin, white blood cell count, and total bilirubin. GNRI, Geriatric Nutritional Risk Index; ALI, Advanced Lung Cancer Inflammation Index; PNI, Prognostic Nutritional Index; OR, Odds Ratio; CI, Confidence Interval.

These findings were corroborated mechanistically via multivariable linear regression utilizing continuous SMI as the dependent variable (Supplementary Table 2). Higher GNRI and ALI scores independently correlated with linear increments in SMI (Model 3, P for trend < 0.001), whereas the PNI model again exhibited a ceiling effect, demonstrating diminished sensitivity in differentiating muscle mass among patients with higher index scores.

### 3.4 Predictive performance and pairwise comparisons

**Figure 5:**
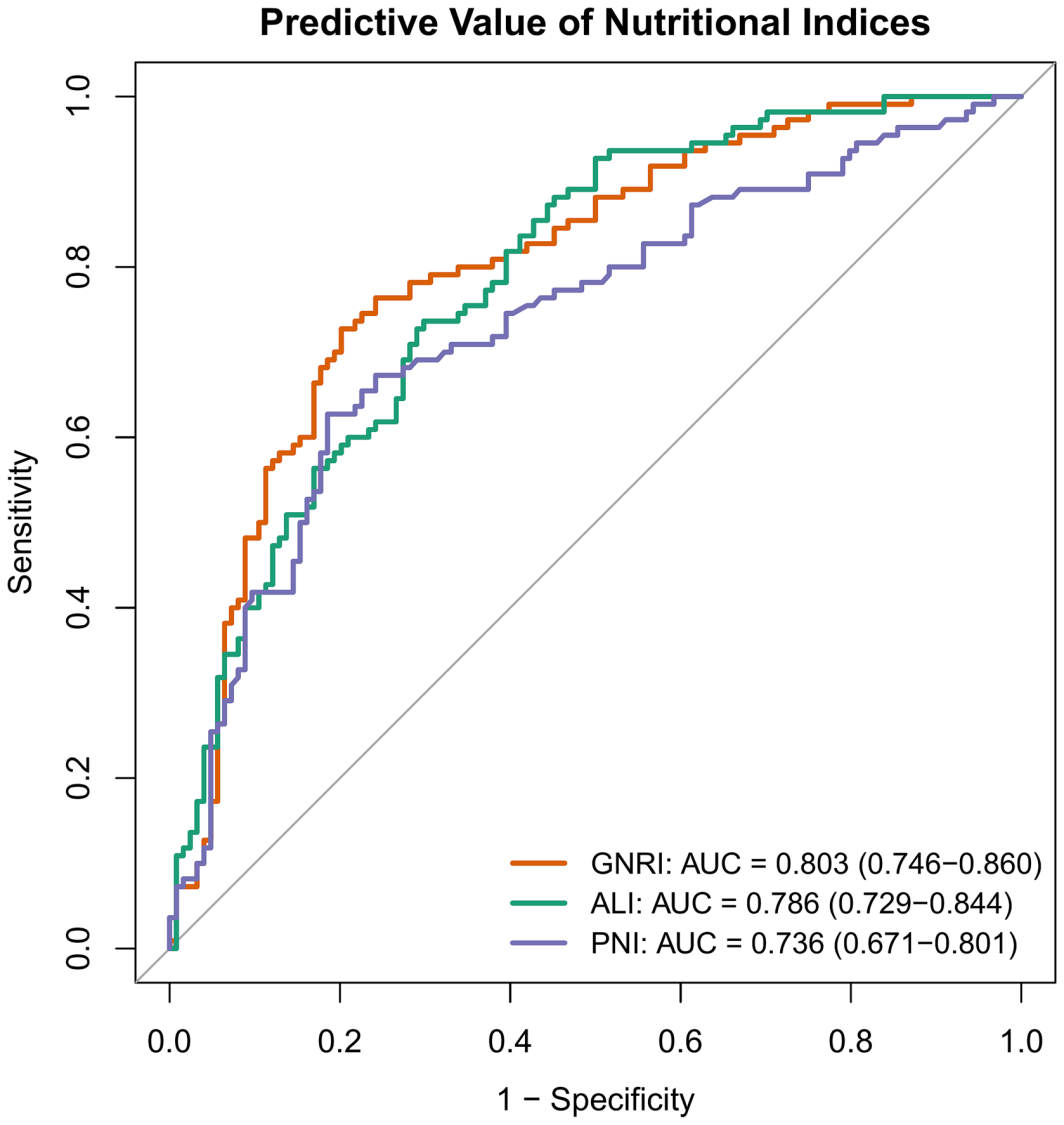
Receiver operating characteristic (ROC) curves of the three nutritional indices for predicting sarcopenia in the training cohort.

Receiver operating characteristic (ROC) analysis was employed to evaluate the discriminative capabilities of the individual nutritional indices (Figure 5; Table 4). The GNRI demonstrated robust predictive accuracy with an AUC of 0.803 (95% CI, 0.746–0.860). Although the GNRI yielded a numerically larger AUC than the ALI (AUC = 0.786, 95% CI: 0.729–0.844), DeLong’s test confirmed that the predictive performance of these two indices was statistically comparable (*p* = 0.597). In contrast, the discriminative performance of the GNRI was significantly superior to that of the PNI (AUC = 0.736, 95% CI: 0.671–0.801; *p* < 0.001).

**Table 4:** Predictive performance and pairwise comparisons of nutritional indices for sarcopenia.

Indices	AUC	95% CI	*p*-value
GNRI	0.803	0.746–0.860	-
ALI	0.786	0.729–0.844	-
PNI	0.736	0.671–0.801	-
GNRI vs. ALI	-	-	0.597
GNRI vs. PNI	-	-	**<0.001**
ALI vs. PNI	-	-	0.109

AUC comparisons were performed using DeLong’s test. Dash (−) indicates the reference line for self-comparison. AUC, Area Under the Curve; CI, Confidence Interval; GNRI, Geriatric Nutritional Risk Index; ALI, Advanced Lung Cancer Inflammation Index; PNI, Prognostic Nutritional Index. The bold values indicate statistical significance (*p* < 0.05).

### 3.5 Development and validation of the predictive nomograms

**Figure 6:**
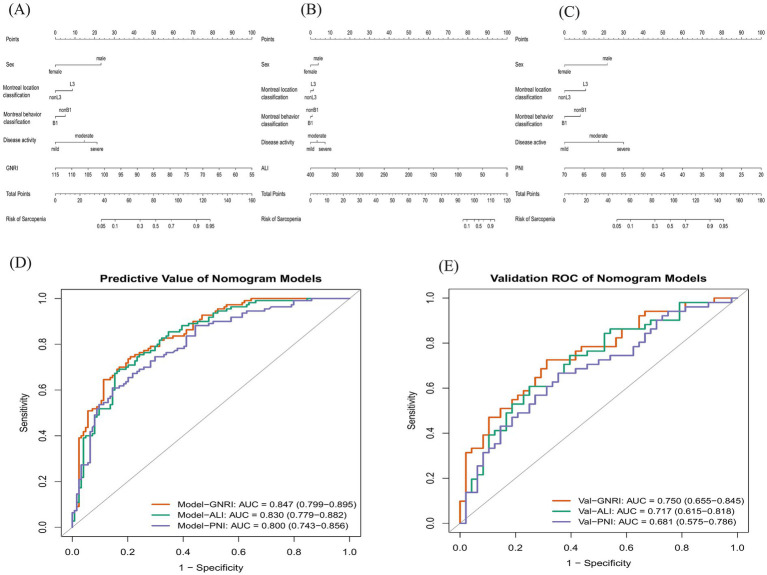
Development and discriminative performance of the predictive nomogram models. Nomograms incorporating clinical features with GNRI **(A)**, ALI **(B)**, and PNI **(C)**. ROC curves comparing the three nomogram models in the training cohort **(D)** and the validation cohort **(E)**.

To facilitate individualized clinical risk estimation, three predictive nomograms were constructed integrating four routine clinical features (sex, Montreal location, Montreal behavior, and disease activity) with each of the nutritional indices (Figures 6A–C).

The Model-GNRI demonstrated superior discrimination, achieving the highest AUC in both the training cohort (AUC = 0.847; Figure 6D) and the internal validation cohort (AUC = 0.750; Figure 6E). Pairwise comparisons (Supplementary Table 3) verified that Model-GNRI significantly outperformed Model-PNI in both cohorts (*p* < 0.05).

**Figure 7:**
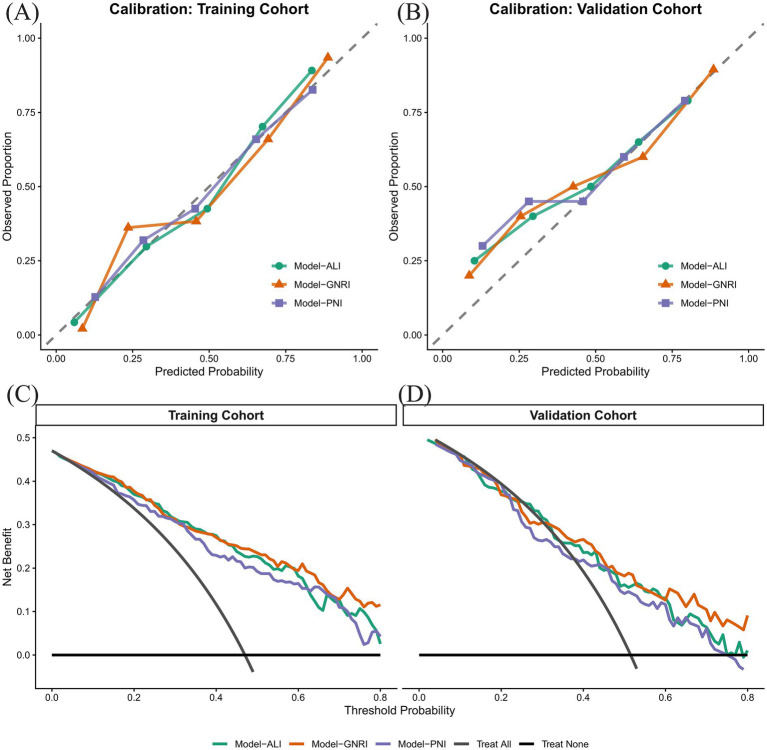
Calibration and clinical utility of the predictive nomogram models. Calibration plots for the nomogram models in the training cohort **(A)** and validation cohort **(B)**. Decision curve analysis (DCA) of the models in the training cohort **(C)** and validation cohort **(D)**.

Calibration plots (Figures 7A,B) revealed high consistency between nomogram-predicted probabilities and actual observations, with Model-GNRI showing the closest alignment to the ideal diagonal line. Furthermore, decision curve analysis (DCA; Figures 7C,D) demonstrated that all predictive models provided positive net clinical benefit compared to default “treat-all” or “treat-none” strategies. Importantly, rather than showing utility only at extreme risk thresholds, Model-GNRI consistently yielded the highest net benefit across a broad and clinically relevant range of threshold probabilities. From a clinical perspective, this indicates that whether a physician adopts a proactive approach (intervening at lower predicted risks) or a more conservative approach (intervening only at higher predicted risks), utilizing the GNRI-based nomogram leads to superior clinical decision-making and optimal allocation of targeted nutritional interventions.

### 3.6 Subgroup analyses and interaction tests

Subgroup analyses were conducted to verify the prognostic stability of the indices across various clinical strata (Supplementary Figure 1). The predictive effect of GNRI remained uniformly significant across all subgroups, including sex, BMI, and disease activity, without any significant interaction (all P for interaction > 0.05). Conversely, significant interaction effects were detected for both ALI (P for interaction = 0.049) and PNI (P for interaction = 0.012) concerning disease activity. These results suggest that while systemic inflammation significantly confounds the predictive stability of ALI and PNI, the GNRI serves as a highly robust biomarker for sarcopenia independent of Crohn’s disease activity flare-ups.

## 4 Discussion

This study provides a head-to-head comparative analysis of three objective nutritional indices (GNRI, ALI, and PNI) for predicting sarcopenia in a well-defined cohort of patients with Crohn’s disease (CD). Our findings indicate that the GNRI demonstrates comparable predictive performance to the ALI, and significantly outperforms the PNI as a predictive biomarker. Based on these results, we developed and internally validated a clinical nomogram integrating the GNRI with routine clinical features (sex, Montreal location, Montreal behavior, and disease activity). This predictive model demonstrated excellent discrimination, adequate calibration, and positive clinical net benefit, offering a practical tool for early sarcopenia risk stratification in patients with CD.

In the present cohort, the prevalence of CT-defined sarcopenia (strictly representing anatomic muscle depletion or myopenia) was 48.3%. While this rate is notably higher than the overall prevalence of functional sarcopenia (typically < 30%) reported in broad, community-based IBD cohorts (26–28), it aligns closely with existing literature utilizing CT-based body composition analysis, which estimates that muscle depletion affects approximately 30 to 60% of patients with IBD (29). Specifically, recent systematic reviews confirm that while strictly defined functional sarcopenia is less common, anatomic myopenia affects up to 42% of adult IBD patients (26), and specific CD cohorts requiring biologic therapies have reported CT-defined sarcopenia rates as high as 45% (30). The substantial muscle depletion observed accurately reflects the severe nutritional and inflammatory burden characteristic of our specific study population. Our cohort exclusively comprised hospitalized patients with CD, the vast majority of whom presented with moderate-to-severe disease activity requiring inpatient care. As extensive literature confirms, the acute activity phase of CD significantly and negatively drives body composition deterioration. Furthermore, a critical methodological strength of this study is the application of population-specific diagnostic thresholds. Rather than applying Western consensus criteria—which may cause substantial misclassification bias due to inherent ethnic differences in body composition—we defined muscle depletion using CT-derived SMI cut-offs (44.77 cm^2^/m^2^ for males and 32.5 cm^2^/m^2^ for females) that were previously established and validated in large-scale Chinese cohorts (25). This tailored approach directly answers the call for standardized, population-based definitions in IBD body composition research, ensuring precise identification within our specific demographic context. The pathogenesis of muscle depletion in CD is multifactorial, driven primarily by chronic systemic inflammation, malabsorption, and altered gut microbiota, all of which disrupt muscle protein synthesis and accelerate catabolism (31–34). Although cross-sectional CT imaging at the L3 level remains the reference standard for quantifying skeletal muscle mass, routine abdominal CT scans strictly for nutritional screening are restricted by radiation exposure, healthcare costs, and the requirement for specialized software (14, 35–38). Consequently, the identification of a reliable, blood-based surrogate marker holds substantial clinical value.

Among the evaluated indices, the GNRI demonstrated a strong predictive value for sarcopenia (AUC = 0.803), reflecting its effective integration of visceral protein status and macroscopic tissue depletion. However, as correctly pointed out during the review process, this performance must be interpreted with caution. Although the GNRI numerically yielded the highest AUC, its predictive accuracy was not statistically superior to that of the ALI (*p* = 0.597). This suggests that while the GNRI provides excellent discriminatory power, the ALI serves as a comparably effective tool for identifying at-risk patients in clinical settings. Furthermore, the GNRI maintained a consistent, linear continuous relationship with the risk of sarcopenia. Originally formulated for the geriatric population, the GNRI mathematically integrates serum albumin with the ratio of actual to ideal body weight. In the context of CD, serum albumin functions as an indicator of both the visceral protein pool and systemic inflammation severity, while the body weight ratio captures chronic macroscopic tissue depletion (39, 40). Subgroup analyses further highlighted the universal applicability of the GNRI. Its predictive value remained highly stable across diverse clinical strata and, crucially, exhibited no significant interaction with CD disease activity (P for interaction > 0.05). This suggests that the GNRI reflects long-term nutritional and muscular reserves rather than fluctuating as a bystander during acute inflammatory flare-ups.

A critical consideration in interpreting our findings is the intrinsic “circularity” or conceptual overlap between the nutritional indices and the outcome of sarcopenia. Since the GNRI, ALI, and PNI are composite measures incorporating components such as albumin and BMI—which are themselves key contributors to muscle health—their “independence” as risk factors is primarily a statistical observation after adjusting for clinical confounders. This intrinsic circularity also accounts for the baseline associations observed in our cohort. Because sarcopenic patients inherently present with depleted foundational components (i.e., significantly lower BMI, albumin, and lymphocytes), the subsequent reduction in their derived composite indices is, to a large extent, an expected mathematical consequence. We acknowledge that these indices represent integrated biological proxies of the nutritional-inflammatory axis rather than entirely independent biological markers. Therefore, their clinical utility lies in their ability to offer a simplified, blood-based reflection of complex nutritional status, even if their components share common pathophysiological roots with the muscle depletion process itself.

Conversely, our analysis elucidated the mechanistic limitations of the PNI and ALI in this specific patient population. Multivariable linear regression revealed a distinct “ceiling effect” for the PNI. While it effectively stratified sarcopenia risk at lower scores, its protective association plateaued in the highest tertile. Since the PNI relies heavily on absolute lymphocyte counts—which are frequently perturbed by immunosuppressive therapies or acute infections inherent to CD—its discriminatory ability diminishes among relatively well-nourished patients (41). Similarly, the performance of both the ALI and PNI was significantly confounded by varying degrees of disease activity, as confirmed by our interaction tests (*p* = 0.049 and 0.012, respectively). Because both indices incorporate volatile systemic inflammatory markers (lymphocytes or the neutrophil-to-lymphocyte ratio), intense acute inflammation in active CD can cause dramatic shifts in leukocyte subpopulations, rendering these indices less representative of the chronic, slow-progressing nature of sarcopenia (42).

To facilitate clinical application, the GNRI was integrated into a predictive nomogram. By combining the GNRI with readily accessible demographic and Montreal classification data—particularly the L3 phenotype, which reflects extensive absorptive surface area damage predisposing patients to severe malabsorption—the nomogram enables individualized risk assessment without additional costs. The decision curve analysis (DCA) confirmed that utilizing this nomogram to guide targeted interventions—such as tailored protein supplementation or resistance training—yields a significantly higher net clinical benefit compared to non-selective strategies, thereby optimizing resource allocation in gastroenterology practices.

The present study has several notable strengths that reinforce the validity of its findings. First, this is the first study to conduct a rigorous head-to-head comparison of these three widely used nutritional indices specifically for sarcopenia in CD, providing definitive clinical guidance rather than isolated index evaluations. Second, we enforced an exceptionally strict set of inclusion and exclusion criteria. By meticulously excluding patients with other severe consumptive diseases, organ failures, and prolonged immobility, we successfully isolated CD-driven muscle depletion from other secondary causes of sarcopenia. Third, the stringent 7-day interval between abdominal CT acquisitions and laboratory blood draws minimized temporal confounding, ensuring the precise synchrony of muscle and nutritional evaluations. Finally, the application of unadjusted restricted cubic splines (RCS) clarified the raw continuous relationships without forced linear assumptions, while the inclusion of an internal validation cohort substantially enhanced the nomogram’s reproducibility.

However, several limitations must be acknowledged and addressed in future research. First, and most importantly, our predictive nomogram was developed and validated internally within a retrospective, single-center cohort. We lack an independent external validation cohort. Given the well-recognized demographic, phenotypic, and therapeutic heterogeneity of CD populations across different institutions, the single-center nature of our data substantially limits the immediate broad generalizability and universal clinical applicability of the proposed model. Furthermore, an inherent selection bias may exist, as our cohort primarily consisted of hospitalized patients who represent a more severe disease spectrum. Therefore, rigorous external validation in large-scale, prospective, multicenter cohorts is essential to confirm the robustness of this nomogram before its widespread adoption in diverse clinical settings. Second, our assessment was based on a single timepoint, whereas sarcopenia and nutritional indices are dynamic parameters that may change substantially following remission induction or biological therapy; the lack of longitudinal data limits our understanding of how variations in the GNRI relate to muscle mass recovery over time. Third, the definition of sarcopenia in this study relied solely on CT-derived muscle mass, without evaluation of muscle composition or functional parameters such as handgrip strength or gait speed, which are important components in current consensus definitions. Finally, potential unmeasured confounders, including detailed dietary intake, levels of physical activity, and cumulative exposure to biological therapies, were not fully accounted for and should be carefully adjusted for in future prospective studies.

## 5 Conclusion

The GNRI serves as a robust surrogate biomarker for sarcopenia in patients with Crohn’s disease, demonstrating excellent predictive performance that is comparable to the ALI and significantly superior to the PNI, alongside high clinical stability. The developed GNRI-based nomogram offers a practical, non-invasive, and accurate screening tool for identifying high-risk patients. The implementation of this model in routine clinical practice may facilitate timely, targeted nutritional and physical interventions, thereby optimizing long-term patient management.

## Data Availability

The datasets presented in this article are not readily available because of patient confidentiality and privacy restrictions. Requests to access the datasets should be directed to the corresponding authors. Requests to access the datasets should be directed to Haiyuan Zhong, 18378916176@163.com.
