# Supplementary material for: Evaluating objective nutritional biomarkers for the prediction of sarcopenia in Crohn’s disease: development and internal validation of a clinical nomogram
Source: Front Nutr. 2026 Jul 9;13:1838106. doi: 10.3389/fnut.2026.1838106 (PMC13394274; doi:10.3389/fnut.2026.1838106)
Supplement: Supplementary file 1 [file Table_1.DOCX]

**Supplementary table 1. Baseline characteristics of the training and validation cohorts**

| **Characteristics** | **Overall (N=333)** | **Training Cohort (n=234)** | **Validation Cohort (n=99)** | **P-value** |
| --- | --- | --- | --- | --- |
| **Sex, n (%)** |  |  |  | 0.714 |
| Male | 229 (68.8) | 159 (67.9) | 70 (70.7) |  |
| Female | 104 (31.2) | 75 (32.1) | 29 (29.3) |  |
| **Age (years)** | 34.86 ± 11.66 | 35.03 ± 11.67 | 34.45 ± 11.69 | 0.679 |
| **BMI (kg/m²)** | 19.04 ± 3.45 | 19.10 ± 3.57 | 18.90 ± 3.18 | 0.64 |
| **Montreal age, n (%)** |  |  |  | 0.759 |
| A2 | 223 (67.0) | 155 (66.2) | 68 (68.7) |  |
| A3 | 110 (33.0) | 79 (33.8) | 31 (31.3) |  |
| **Montreal location, n (%)** |  |  |  | 1 |
| L3 | 213 (64.0) | 150 (64.1) | 63 (63.6) |  |
| non-L3 | 120 (36.0) | 84 (35.9) | 36 (36.4) |  |
| **Montreal behavior, n (%)** |  |  |  | 0.717 |
| B1 | 185 (55.6) | 128 (54.7) | 57 (57.6) |  |
| non-B1 | 148 (44.4) | 106 (45.3) | 42 (42.4) |  |
| **Disease activity, n (%)** |  |  |  | 0.436 |
| Mild | 28 (8.4) | 21 (9.0) | 7 (7.1) |  |
| Moderate | 284 (85.3) | 196 (83.8) | 88 (88.9) |  |
| Severe | 21 (6.3) | 17 (7.3) | 4 (4.0) |  |
| **Smoking history, n (%)** |  |  |  | 0.95 |
| No | 248 (74.5) | 175 (74.8) | 73 (73.7) |  |
| Yes | 85 (25.5) | 59 (25.2) | 26 (26.3) |  |
| **Drinking history, n (%)** |  |  |  | 0.496 |
| No | 256 (76.9) | 177 (75.6) | 79 (79.8) |  |
| Yes | 77 (23.1) | 57 (24.4) | 20 (20.2) |  |
| **Surgery history, n (%)** |  |  |  | 0.326 |
| No | 292 (87.7) | 202 (86.3) | 90 (90.9) |  |
| Yes | 41 (12.3) | 32 (13.7) | 9 (9.1) |  |
| **Perianal complication, n (%)** |  |  |  | 0.338 |
| No | 249 (74.8) | 171 (73.1) | 78 (78.8) |  |
| Yes | 84 (25.2) | 63 (26.9) | 21 (21.2) |  |
| **WBC (×10⁹/L)** | 8.05 ± 3.78 | 7.94 ± 3.90 | 8.30 ± 3.46 | 0.427 |
| **Hemoglobin (g/L)** | 113.28 ± 24.40 | 112.30 ± 24.97 | 115.60 ± 22.94 | 0.26 |
| **Platelets (×10⁹/L)** | 374.79 ± 137.84 | 384.18 ± 143.36 | 352.58 ± 121.64 | 0.056 |
| **Neutrophils (×10⁹/L)** | 5.77 ± 3.59 | 5.68 ± 3.77 | 5.96 ± 3.13 | 0.521 |
| **Lymphocytes (×10⁹/L)** | 1.38 ± 0.60 | 1.35 ± 0.60 | 1.46 ± 0.60 | 0.122 |
| **Total bilirubin (umol/L)** | 8.54 ± 10.66 | 8.87 ± 12.45 | 7.76 ± 3.90 | 0.387 |
| **Albumin (g/L)** | 37.31 ± 6.60 | 37.10 ± 6.51 | 37.82 ± 6.81 | 0.367 |
| **SMI (cm²/m²)** | 41.16 ± 8.64 | 41.35 ± 8.60 | 40.72 ± 8.78 | 0.546 |
| **Sarcopenia, n (%)** |  |  |  | 0.527 |
| No | 172 (51.7) | 124 (53.0) | 48 (48.5) |  |
| Yes | 161 (48.3) | 110 (47.0) | 51 (51.5) |  |
| **GNRI** | 90.38 ± 12.49 | 90.03 ± 12.37 | 91.22 ± 12.80 | 0.428 |
| **ALI** | 24.70 ± 27.92 | 25.60 ± 31.60 | 22.56 ± 16.11 | 0.364 |
| **PNI** | 44.23 ± 7.93 | 43.85 ± 7.98 | 45.12 ± 7.78 | 0.181 |

Continuous variables are presented as mean ± standard deviation (SD), and categorical variables are presented as frequencies and percentages (%). P values comparing the training and validation cohorts were calculated using the Student’s t-test for continuous variables and the Chi-square test for categorical variables. All P values > 0.05 indicate a well-balanced distribution of baseline characteristics between the two cohorts.

**Abbreviations:** BMI, Body Mass Index; WBC, White Blood Cell; SMI, Skeletal Muscle Index; GNRI, Geriatric Nutritional Risk Index; ALI, Advanced Lung Cancer Inflammation Index; PNI, Prognostic Nutritional Index.

**Supplementary table 2. Association between nutritional indices and Skeletal Muscle Index (SMI) across different models**

| **Variables** | | **SMI (mean ± SD)** | **Model 1** |  | **Model 2** |  | **Model 3** |  |
| --- | --- | --- | --- | --- | --- | --- | --- | --- |
|  |  |  | **β (95% CI)** | ***P-*value** | **β (95% CI)** | ***P-*value** | **β (95% CI)** | ***P-*value** |
| **GNRI** | Continuous (per 1 unit) | - | 0.386 (0.324, 0.448) | <0.001 | 0.344 (0.290, 0.398) | <0.001 | 0.305 (0.235, 0.374) | <0.001 |
|  | T1 (ref) | 35.53 ± 6.90 | - | - | - | - | - | - |
|  | T2 | 41.53 ± 7.74 | 5.994 (4.033, 7.954) | <0.001 | 5.168 (3.505, 6.830) | <0.001 | 3.863 (2.046, 5.679) | <0.001 |
|  | T3 | 46.43 ± 7.60 | 10.894 (8.933, 12.854) | <0.001 | 9.674 (7.970, 11.379) | <0.001 | 7.640 (5.489, 9.791) | <0.001 |
|  | *P* for trend |  |  | <0.001 |  | <0.001 |  | <0.001 |
| **ALI** | Continuous (per 1 unit) | - | 0.108 (0.077, 0.139) | <0.001 | 0.086 (0.059, 0.113) | <0.001 | 0.061 (0.034, 0.088) | <0.001 |
|  | T1 (ref) | 36.90 ± 7.91 | - | - | - | - | - | - |
|  | T2 | 40.75 ± 7.47 | 3.850 (1.777, 5.923) | <0.001 | 3.069 (1.325, 4.812) | <0.001 | 2.651 (0.868, 4.433) | 0.004 |
|  | T3 | 45.84 ± 8.15 | 8.938 (6.865, 11.011) | <0.001 | 7.743 (5.943, 9.544) | <0.001 | 6.508 (4.426, 8.590) | <0.001 |
|  | *P* for trend |  |  | <0.001 |  | <0.001 |  | <0.001 |
| **PNI** | Continuous (per 1 unit) | - | 0.469 (0.363, 0.576) | <0.001 | 0.391 (0.296, 0.486) | <0.001 | 0.235 (0.115, 0.354) | <0.001 |
|  | T1 (ref) | 36.43 ± 7.80 | - | - | - | - | - | - |
|  | T2 | 42.84 ± 8.02 | 6.404 (4.299, 8.508) | <0.001 | 5.341 (3.555, 7.128) | <0.001 | 3.348 (1.504, 5.193) | <0.001 |
|  | T3 | 44.22 ± 8.09 | 7.786 (5.681, 9.891) | <0.001 | 6.377 (4.549, 8.205) | <0.001 | 2.837 (0.640, 5.034) | 0.012 |
|  | *P* for trend |  |  | <0.001 |  | <0.001 |  | 0.015 |

Model 1: Crude model (unadjusted).

Model 2: Adjusted for sex, Montreal age, Montreal location, Montreal behavior, disease activity, smoking history, drinking history, intestinal surgery history, and perianal complication.

Model 3: Adjusted for sex, Montreal age, Montreal location, Montreal behavior, disease activity, smoking history, drinking history, intestinal surgery history, and perianal complication, hemoglobin, white blood cell count, and total bilirubin.

β represents the unstandardized regression coefficient, indicating the mean change in SMI.

**Abbreviations:** CI, Confidence Interval; GNRI, Geriatric Nutritional Risk Index; ALI, Advanced Lung Cancer Inflammation Index; PNI, Prognostic Nutritional Index; SD, Standard Deviation.

**Supplementary table 3. Predictive performance and pairwise comparisons of the nomogram models**

| **Models** | **AUC (95% CI)** | **P-value vs. Model-GNRI** | **P-value vs. Model-ALI** | **P-value vs. Model-PNI** |
| --- | --- | --- | --- | --- |
| **Training Cohort** |  |  |  |  |
| Model-GNRI | 0.847 (0.799–0.895) | - | 0.479 | <0.001 |
| Model-ALI | 0.830 (0.779–0.882) | 0.479 | - | 0.155 |
| Model-PNI | 0.800 (0.743–0.856) | <0.001 | 0.155 | - |
| **Validation Cohort** |  |  |  |  |
| Model-GNRI | 0.750 (0.655–0.845) | - | 0.412 | 0.011 |
| Model-ALI | 0.717 (0.615–0.818) | 0.412 | - | 0.351 |
| Model-PNI | 0.681 (0.575–0.786) | 0.011 | 0.351 | - |

All models were adjusted for sex, Montreal location, Montreal behavior, and disease activity.

AUC comparisons were performed using DeLong's test.

Dash (-) indicates the reference line for self-comparison.

Abbreviations: AUC, Area Under the Curve; CI, Confidence Interval; GNRI, Geriatric Nutritional Risk Index; ALI, Advanced Lung Cancer Inflammation Index; PNI, Prognostic Nutritional Index.


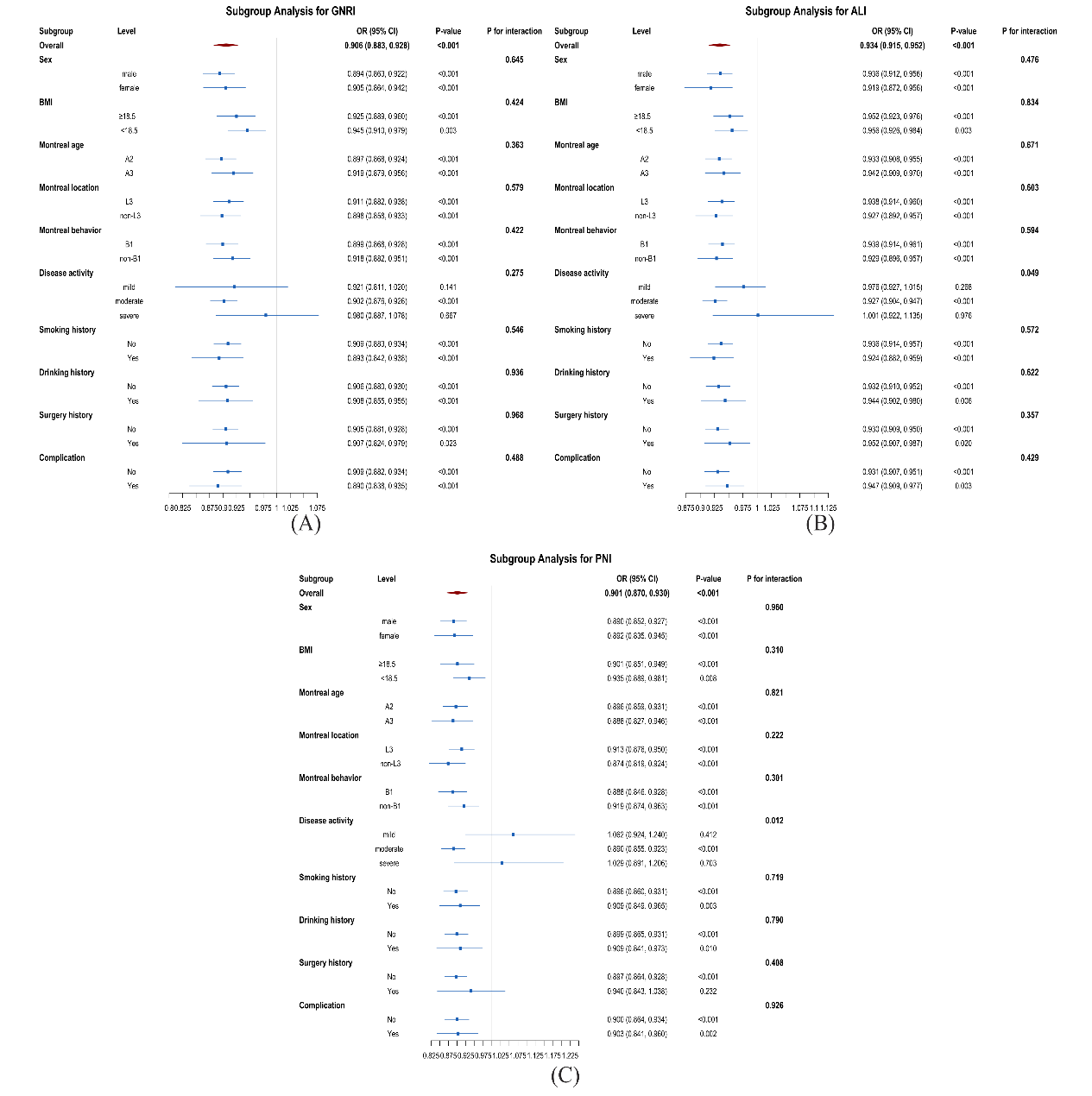


**Supplementary figure 1. Subgroup analyses and interaction tests of the nutritional indices for predicting sarcopenia.**

Forest plots displaying the odds ratios (ORs) and 95% confidence intervals (CIs) of GNRI (A), ALI (B), and PNI (C) across various clinical and demographic subgroups.
